# WaveletQuant, an improved quantification software based on wavelet signal threshold de-noising for labeled quantitative proteomic analysis

**DOI:** 10.1186/1471-2105-11-219

**Published:** 2010-04-29

**Authors:** Fan Mo, Qun Mo, Yuanyuan Chen, David R Goodlett, Leroy Hood, Gilbert S Omenn, Song Li, Biaoyang Lin

**Affiliations:** 1 Systems Biology Division, Zhejiang-California Nanosystems Institute (ZCNI) of Zhejiang University, Zhejiang University Huajiachi Campus, 268 Kaixuan Road, Hangzhou 310029, China; 2 Department of Mathematics, College of Science, Zhejiang University Yuquan Campus, 38 Zheda Road, Hangzhou 310027, China; 3 Department of Medicinal Chemistry, University of Washington, Seattle, Washington, USA; 4 The Institute for Systems Biology, Seattle, Washington, USA; 5 Center for Computational Medicine and Biology, University of Michigan, Ann Arbor, MI 48109, USA; 6 Swedish Neuroscience Institute, Swedish Medical Center, Seattle, WA 98122, USA; 7 Dept. of Urology, University of Washington, Seattle, WA 98195, USA

## Abstract

**Background:**

Quantitative proteomics technologies have been developed to comprehensively identify and quantify proteins in two or more complex samples. Quantitative proteomics based on differential stable isotope labeling is one of the proteomics quantification technologies. Mass spectrometric data generated for peptide quantification are often noisy, and peak detection and definition require various smoothing filters to remove noise in order to achieve accurate peptide quantification. Many traditional smoothing filters, such as the moving average filter, Savitzky-Golay filter and Gaussian filter, have been used to reduce noise in MS peaks. However, limitations of these filtering approaches often result in inaccurate peptide quantification. Here we present the WaveletQuant program, based on wavelet theory, for better or alternative MS-based proteomic quantification.

**Results:**

We developed a novel discrete wavelet transform (DWT) and a 'Spatial Adaptive Algorithm' to remove noise and to identify true peaks. We programmed and compiled WaveletQuant using Visual C++ 2005 Express Edition. We then incorporated the WaveletQuant program in the **Trans-Proteomic Pipeline (TPP)**, a commonly used open source proteomics analysis pipeline.

**Conclusions:**

We showed that WaveletQuant was able to quantify more proteins and to quantify them more accurately than the ASAPRatio, a program that performs quantification in the TPP pipeline, first using known mixed ratios of yeast extracts and then using a data set from ovarian cancer cell lysates. The program and its documentation can be downloaded from our website at http://systemsbiozju.org/data/WaveletQuant.

## Background

Quantitative proteomics technologies have been developed to comprehensively identify and quantify proteins in two or more complex samples [1-4]. There are three ways to perform quantitative proteomic analysis: a) the spectral counting method that counts the number of fragment ion spectra for a particular peptide [5]; b) differential stable isotope labeling, in which quantified peptides differ by the mass shifts introduced by the stable isotopes used [6]; and c) label-free quantification that quantifies the precursor ion signal intensities across different LC-MS runs [7-9].

Quantification using the differential stable isotope labeling method is one of the methods for quantification of two or more samples within a single experiment. The technique is based on use of stable isotopes to differentially label proteins or peptides, and on use of mass spectrometry to compare the relative abundance of the proteins in different samples. Over the years, many stable isotope tagging approaches have been developed, which include the ICAT [6], ITRAQ [10], and SILAC [11] approaches. In addition, numerous quantification software were developed, including XPRESS [6], ASAPRatio [12], MSQuant http://msquant.sourceforge.net/, ZoomQuant [13], STEM [14], Multi-Q [15], i-tracker [16], Libra [17], maxQuant [18], muxQuant [19], HTAPP (high-throughput autonomous proteomic pipeline) [20], msInspect [21], the APEX Quantitative Proteomics Tool [22], MASIC [23], and Census [24].

In our quantitative proteomics analysis, we found that errors associated with ratios calculated by the ASAPRatio increased proportionally with the relative abundance ratios of the two isotopic partners. Several factors might have contributed to the increase of relative errors. We found one of the factors to be background noise that was not completely removed by the Savitzky-Golay smooth filtering method.

Wavelets are mathematical functions that divide a given function or a continuous-time signal into different frequency components, and then study each component with a resolution matched to its scale [25,26]. They have advantages over traditional Fourier transforms in analyzing data for which signals have discontinuities and sharp peaks, and in deconstructing and reconstructing signals more accurately [27].

Various programs integrating wavelet transforms have been developed for analyzing various types of proteomics data, such as MALDI, SELDI-TOF and LC/MS. Yang et al. compared five smoothing methods used in peak detection algorithms for MALDI mass spectrometry data analysis [28]. They found that the wavelet smoothing performed best among the five smoothing methods: moving average filter, Savitzky-Golay filter, Gaussian filter, Kaiser window, and wavelet based filters [28]. Du et al. showed that a continuous wavelet transform (CWT)-based peak detection algorithm enhances the effective signal-to-noise ratio in SELDI-TOF spectra; it could identify both strong and weak peaks while keeping false positive rates low [29]. Randolph and Yasui applied a translation-invariant wavelet analysis to perform multiscale decomposition, feature extraction and quantification for MALDI-TOF spectra [30]. Alexandrov et al. developed the MALDIDWT program for analyzing serum protein profiles for biomarker discovery [31]. Lange et al. used wavelet techniques to develop a mass spectrometer-independent peak-picking algorithm as an alternative to vendors' peak-picking software bundled with mass spectrometers [32]. Schulz-Trieglaff et al. developed an algorithm that uses a mother wavelet to mimic the distribution of isotopic peak intensities [33]. The latter two algorithms by Lange et al. and Schulz-Trieglaff et al. were further implemented in OpenMS software [34]. Zhang et al. used an undecimated wavelet transform to remove random noise for prOTOF MS data, which does not require a priori knowledge of protein masses[35]. Using metabolomics data as examples, Tautenhahn et al. developed a new feature detection algorithm centWave for high-resolution LC/MS data sets applying continuous wavelet transformation and optional Gauss-fitting in the chromatographic domain[36].

Wavelet theory has also been applied to MS data to reduce data dimension or to reduce computation time. For example, Hussong et al. implemented a feature finding algorithm based on a hand-tailored adaptive wavelet transform that drastically reduces the computation time in mass spectrometry data analysis [37]. Liu et al. used the wavelet detail coefficients to characterize features and reduce the dimensionality of MS data [38].

In this manuscript, we report development of a new wavelet transform algorithm for improved quantitative proteomics analysis. We demonstrate that our approach has an improved ability to smooth isotopic peaks and remove background noise when compared with approaches using other smoothing methods.

### Implementation

#### Technical details of the development of the WaveletQuant program

The wavelet transform is an excellent tool for signal processing because of its de-noising ability; one can obtain multi-resolution decomposition of signals, while retaining their local characteristic details.

The first step in the wavelet transform method is to choose a proper threshold to de-noise signals. The principle of wavelet-based de-noising is to recognize the noise from the high frequency part of wavelet coefficients. Those coefficients that are less than the threshold are set to zero. Other coefficients are preserved. Then we reconstruct the de-noised signals using the new coefficients. As indicated in the Results presented below, setting the wavelet coefficients of noise to zero while at the same time preserving the wavelet coefficients of signals is critical for a successful wavelet transform. Choosing an optimal threshold is the key to retaining maximal true signals while reducing as much noise as possible.

Given a measured signal *x(t) *with a Gaussian white noise *n(t) *can be presented by the following formula:(1)

The method is composed of three components: (i) the discrete wavelet transform (DWT) of signal *x(t)*; (ii) setting the threshold for the wavelet coefficients on each scale; and (iii) obtaining de-noised signals by inverse wavelet transform based on the threshold wavelet coefficients. A more detailed description of the wavelet transform process is shown in Additional file 1.

We then adopted the universal threshold T proposed by Donoho and Johnstone [39] to remove Gaussian white noise, described as follows:(2)

where N is the length of signal *x(t)*, *σ *is the noise level, and MAD is the median absolute deviation estimated in the first scale. The factor *0.6745 *in the denominator rescales the numerator so that is a suitable estimator for the standard deviation for Gaussian white noise. Significant wavelet coefficients could be derived by setting a threshold rule. There are hard and soft threshold rules, each with its advantages and disadvantages.(3)

The hard threshold can preserve local characteristics, but the reconstructed signals are not very smooth. The soft threshold can obtain a smoothed curve, but it always distorts the signal. In this paper, we combined the strengths of the two methods, and developed a new rule:(5)

where 0 <*λ *< 1. When *λ *is zero, it returns to the hard threshold. When *λ *is one, it returns to the soft threshold. We set *λ *= [0.1, 0.4]. The procedure of wavelet-based de-noising is given as:(6)

Xu et al. [40] proposed a spatially selective noise filtration technique. They declared that the singularity of a signal should have a large peak value in different scales, while noise should have fading energy with increasing scales. Inspired by their work, we developed a 'Spatial Adaptive Algorithm' to identify true peaks (a more detailed description of the method is presented in Additional file 1).

Assuming the largest scale of decomposition is *J*, *Wf*(*j, n*)denotes DWT coefficient of signal *f *at position *n *in scale *j*. We denoted the correlation of bordered scales as follows:(7)

where *l *represents the scale and *j *<*J *- *l *+ 1. As the singularity of signals increases along with increasing scales, bordered points affect each other in detailed scales. We chose *l *= 2 to compute the correlation:(8)

where *Corr*_2_(*j, n*) is denoted as correlation coefficient of the position *n *in scale *j*.

To make correlation coefficient and wavelet coefficient more comparable, we defined the correlation coefficient uniformly:

And:(9)

Then we compared *NewCorr*_2_(*j*, *n*) with *Wf*(*j*, *n*) to obtain the edges of important signals. In summary, by multiplying wavelet coefficients of bordered scales, we computed a correlation coefficient to suppress the noise and to strengthen the signal. Our algorithm improved the identification of real signals and the orientational precision of the identified signals.

#### Software implementation

**Figure 1:**
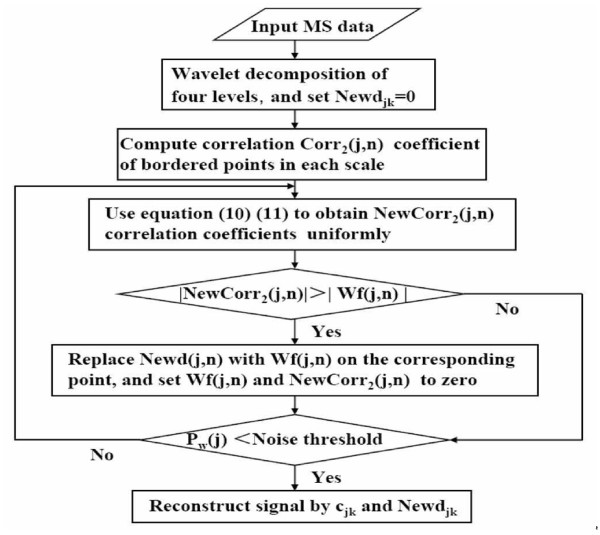
**WaveletQuant program flow chart**. Equations 10 and 11 are shown in additional file 1.

We programmed and compiled WaveletQuant using Visual C++ 2005 Express Edition. A program flow chart is shown in Figure 1. We evaluated peptide abundances by reconstructing a raw single-ion chromatogram over a chromatographic elution period. Then the wavelet algorithms we described in the previous sections were applied to obtain an adjusted chromatogram peak area. Signals inside the peak region were decomposed into four levels and correlation coefficients of bordered points in each scale were calculated. Next, we recursively computed new correlation coefficients and modified them by comparing them with *Wf*(*j*, *n*). According to these new values, a noise threshold was re-calculated. If the wavelet coefficients were less than the noise threshold, they were set to zero. The remaining wavelet coefficients were considered signals. Finally, we reconstructed the de-noised signals and used the de-noised peak areas to calculate peptide abundance. Because the Trans-Proteomic Pipeline (TPP) http://tools.proteomecenter.org/software.php is a commonly used open source proteomics analysis pipeline, we decided to build our program into the TPP. We compiled a new TPP package by replacing the ASAPRatio program with the WaveletQuant program. The package and its documentation can be downloaded from our website at http://www.zcni.zju.edu.cn/en/WaveletQuant_for_Quantitative_Proteomics/waveletquant.html or http://systemsbiozju.org/data/WaveletQuant.

## Result

### Application of the WaveletQuant program to data generated using yeast extracts mixed in known ratios

We compared the performance of our WaveletQuant program with the ASAPRatio program using a dataset generated by mixing different ratios of yeast cell extract grown in heavy vs. light isotopic media. Proteins were mixed in the following ratio 1:2, 1:1.5, 1:1, 1.5:1, 2:1, and then analyzed by LTQ-MS.

**Figure 2:**
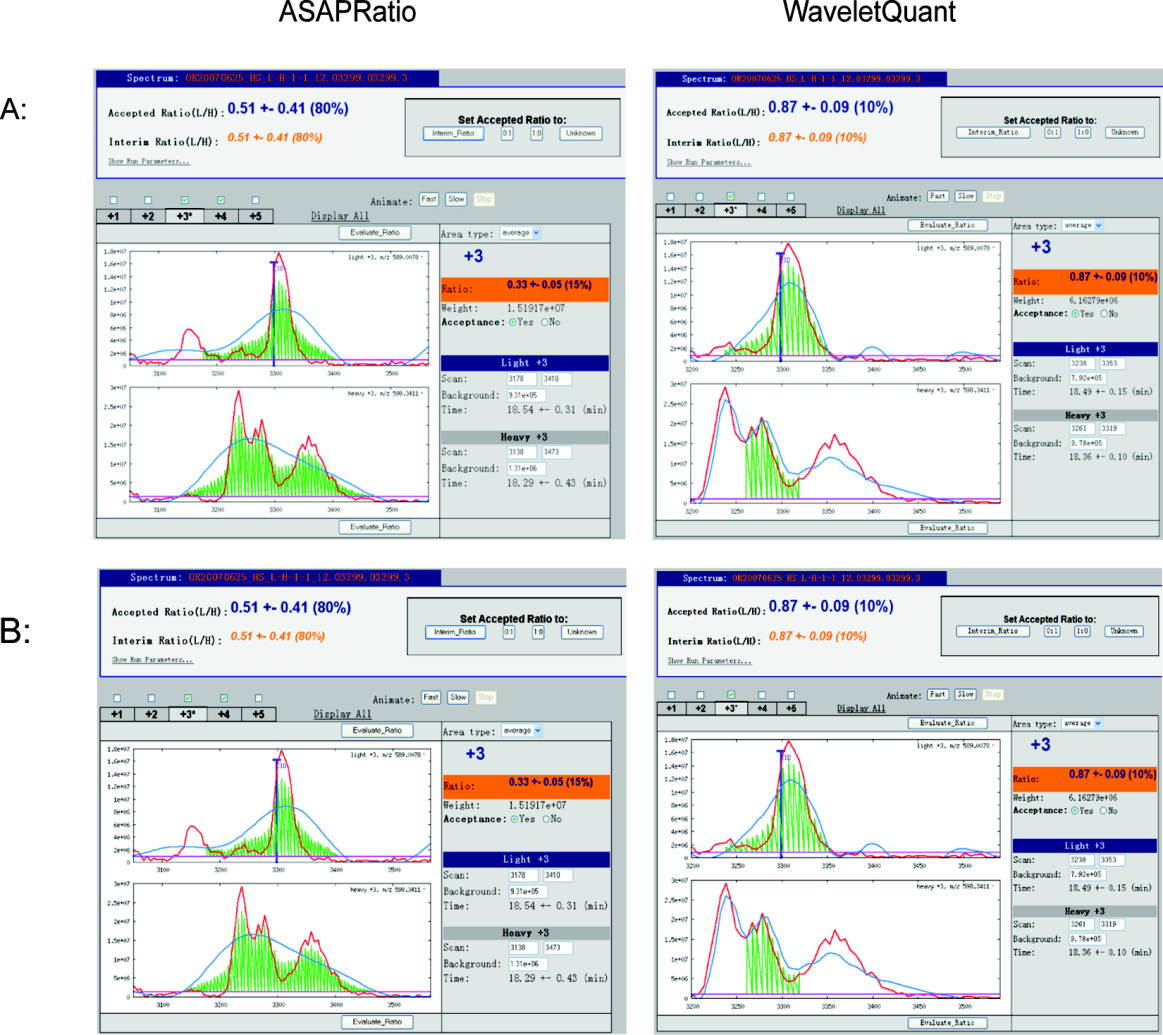
**Comparison of the quantification performance of the WaveletQuant and the ASAPRatio for yeast extracts mixed in 1:1 ratio**. A: Comparison of the quantification performance of yeast extracts mixed in 1:1 ratio between the WaveletQuant (right) and the ASAPRatio (left). The MS spectra OR20070625_HS_L-H-1-1_12.03299.03299.3 (+3 charge state) are illustrated. LC-MS chromatograms of the isotopically light and heavy peptide partners are shown. Raw chromatograms are plotted in red, smoothed chromatograms in blue, areas used for calculating abundance ratio of the charge state in green, and backgrounds in cyan. On the top is peptide abundance ratio. On the right are start and end scan numbers, background, elution time of the isotopically light and heavy peptide partners, acceptance, abundance ratio, and weight of the charge states. Users may change scan numbers, background levels, and acceptance of the charge state. B: Comparison of the quantification performance of yeast extracts mixed in 1:1 ratio between the WaveletQuant (right) and the ASAPRatio (left). MS spectra OR20070625_HS_L-H-1-1_12.10969.10969.2 (+2 charge state) are illustrated.

**Figure 3:**
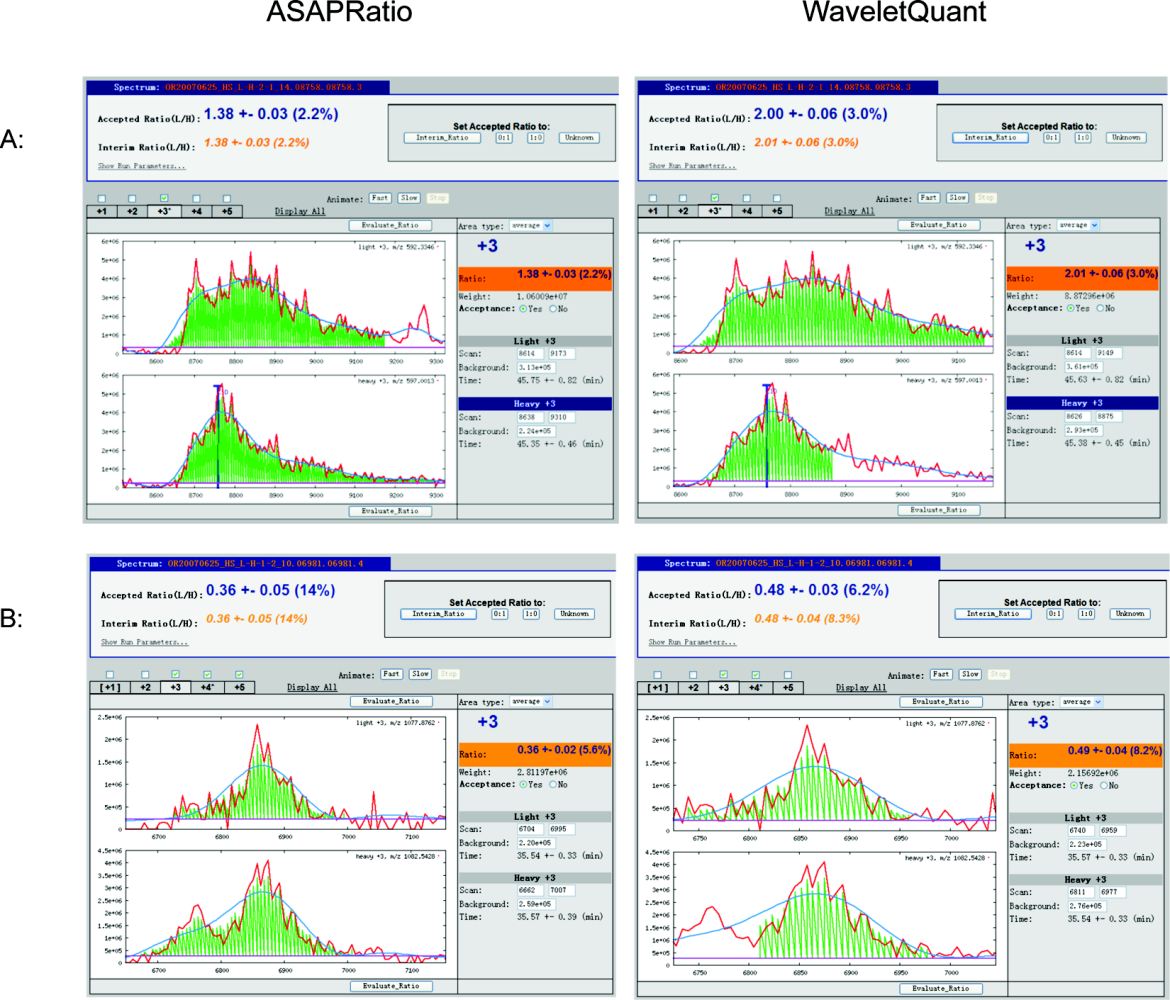
**Comparison of the quantification performance of the WaveletQuant and the ASAPRatio for yeast extracts mixed in 2:1 ratio**. A: Comparison of the quantification performance of yeast extracts mixed in 2:1 ratio between the WaveletQuant (right) and the ASAPRatio (left). MS spectra OR20070625_HS_L-H-4-1_15.10440.10440.3 (+2 charge state) are illustrated. B: Comparison of the quantification performance of yeast extracts mixed in 1:2 ratio between the WaveletQuant (right) and the ASAPRatio (left). MS spectra OR20070625_HS_L-H-1-2_10.06981.06981.4 (+3 charge state) are illustrated.

We found that the WaveletQuant program was able to perform curve fitting for respective chromatogram pairs and to quantify more accurately the difference of mixed yeast extracts than the original ASAPRatio program. Figures 2 and 3 show several examples where the WaveletQuant program performed better than the ASAPRatio program, which uses the Savitzky-Golay filter for denoising. The WaveletQuant program achieved more accurate quantification. An advantage of our program is the ability to separate a high peak from an overlapping low peak. The ASAPRatio program tends to merge the two peaks into one and quantified it. However, the low peak can be noise or a signal from another peptide that eluted immediately following the peptide being analyzed. The most obvious examples are those shown in Figures 2 and 3. In Figure 2A and 3B, the two peaks in the heavy peptide (bottom panel) were regarded as one peak by ASAPRatio program. However, the WaveletQuant program was able to separate the two peaks. For the peptide shown in Figure 2B, ASAPRatio failed to quantify it, but WaveletQuant did find the correct peaks and was able to quantify it. Figure 3A showed a subtle example where the 2^nd ^peak could often be mistaken as from the first peak. In Figure 3A, for the heavy labeled peptide (bottom half of the two panels), our program was able to separate two overlapped peaks, while the ASAPRatio regarded it as one peak. The ASAPRatio therefore over quantified the heavy labeled peptides. Additional examples were shown in Additional file 2, 3 and 4.

The protein abundance ratios for all proteins obtained by the new algorithm and old algorithm of yeast extract mixtures are summarized in Table 1. The ratios were calculated by averaging all unique peptides' ratios after recursively eliminating outliers by calculating the average number A and +- √A's (square root of A) range (except in the first round, they were eliminated by choosing the median m and +- √m's range). We found that relative errors of the ratios obtained by the WaveletQuant method were much less (from 1 to 27% for different known mixed ratios) than those obtained from the ASAPRatio program (from 20 to 52%) (Table 1), suggesting that wavelet-based signal threshold de-noising is more efficient and more precise than the ASAPRatio program.

**Table 1:** Comparison of quantification results between the ASAPRatio and the WaveletQuant programs.

Light/Heavy ratio	Mixed ratio	ASAPRatio ratio	ASAPRatio Relative error (%)	WaveletQuant ratio	WaveletQuant Relative error (%)
L/H	0.5	0.73	47	0.39	21
L/H	0.67	0.85	27	0.64	3
L/H	1	1.51	50	1.09	9
L/H	1.5	1.2	20	1.48	1
L/H	2	0.95	52	2.15	7

### Application of the WaveletQuant program to data generated by ICAT for ovarian cancer cell lines

We have previously conducted quantitative proteomics studies comparing cisplatin-resistant ovarian cancer cells with cisplatin-sensitive cancer cells [41]. Using the RAW data of the cytosolic fractions, we compared TPP with ASAPRatio and TPP with WaveletQuant implementation. We found that TPP with the ASAPRatio was able to quantify the protein expression of 226 proteins, while TPP with WaveletQuant quantified 222 proteins, and 204 proteins were quantified by both algorithms. The total number of proteins quantified combining both programs is 245, which is about 10% more than using either program alone. We found that the average standard deviation for the ratios of quantification were 0.57 for TPP with ASARatio and 0.47 for TPP with WaveletQuant. Thus WaveletQuant appears to have better accuracy for quantification than the ASAPRatio.

## Discussion

We developed a new software for quantitative proteomics using the wavelet transform. Mass spectrometry data are usually noisy. In order to better quantify mass spectrometry data, smoothing filters, such as the moving average filter, Gaussian filter Butterworth low-pass filter, and Savitzky-Golay filter can be used to reduce the noise in MS peaks. The moving average filter was used in the MZmine program [42]. The Gaussian filter was used by the local maximum search (LMS) program, which was developed for SELDI MS data analysis [43]. The smoothing used in the XPRESS program was performed with the Butterworth low-pass filter http://www.qsl.net/kp4md/butrwrth.htm, for which low-frequency excitation signal components down to and including the current ones are transmitted, while high-frequency components, up to and including infinite ones, are blocked. The ASAPRatio program uses the Savitzky-Golay method [12], which performs a least squares fit of a small set of consecutive data points to a polynomial and then takes the central point of the fitted polynomial curve as the output. The Savitzky-Golay smoothing tends to preserve features of the distribution such as relative maxima, minima, and width; this is its main advantage as these features are often 'flattened' by other smoothing methods (e.g. moving averages). However, as we showed in Results, a disadvantage of the Savitzky-Golay filter is that it smoothes signals by increasing window sizes and lowering filter frequencies; thus, the smoothed shape could create poor representations of true signals and generate inaccurate quantification. We found that wavelet smoothing is better than the Savitzky-Golay filter used with ASAPRatio. Yang et al. similarly found that wavelet smoothing performed better than moving average filter, Savitzky-Golay filter, Gaussian filter, and Kaiser window [28].

In addition, we implemented orthogonal wavelets to decompose signals in our WaveletQuant program. The wavelet transform is different from that used by Lange et al. and Schulz-Trieglaff et al. [32,33]. Many wavelets could be chosen to perform wavelet transform, including Daubechies' orthogonal and bi-orthogonal wavelets, Gaussian wavelets and coiflets [25]. Each wavelet has its own advantage depending on wavelet shapes and wavelet widths. The orthogonal wavelet can keep the energy (i.e. sum of squares of coefficients, usually referred to as "energy" in the signal processing field) of a signal unchanged. We have therefore selected the orthogonal wavelet transform for our MS data analysis.

We implemented two methods. First, by combining the advantage of hard threshold and soft threshold, we developed the wavelet-based signal threshold de-noising algorithm to distinguish signals from noise in MS data. Second, we developed the spatial adaptive algorithm, which not only was effective in removing high frequency noise but also was effective for low frequency de-noising. Combining these two algorithms, our WaveletQuant program performs better than the ASAPRatio program on the datasets from yeast that we tested (Figures 2 and 3). Finally, in a test using high throughput proteomics data generated from cell lysates in an ovarian cancer study, we found that the ratios obtained by our program have lower overall standard deviation than that obtained by the ASAPRatio.

Of note, we also mixed proteins in 1:4 and 4:1 ratios and analyzed them by LTQ-MS. However, due to the limited dynamic range of the routine LC/MS that we performed, the average ratios calculated by both the ASAPRatio and the WaveletQuant programs were far-off from the original mixed ratios, with large standard deviation. This is not surprising as the mixed ratios are outside the dynamic range of a routine LC/MS analysis, which Canterbury et al. estimated to be 0.5 to 2.5 in a systematic analysis [44]. This result also suggests that our WaveletQuant program did not improve the dynamic range of the quantification. Another possibility is that the experiment failed due to unknown reasons. Therefore, we have not included the data in this report.

Finally, we have implemented our wavelet transform algorithm and developed the WaveletQuant program. As the TPP pipeline is widely used in proteomic data analysis, we incorporated WaveletQuant software into the TPP pipeline http://tools.proteomecenter.org/TPP.php. Users can employ the WaveletQuant- implemented TPP pipeline as an alternative to the standard TPP pipeline.

## Conclusions

We have developed an improved and/or alternative program for quantitative proteomics analysis, which is implemented in the standard TPP pipeline for the convenience of users.

## Availability and requirements

**• Project name: **WaveletQuant

**• Project home page: **http://www.zcni.zju.edu.cn/en/WaveletQuant_for_Quantitative_Proteomics/waveletquant.html or http://systemsbiozju.org/data/WaveletQuant.

**• Operating systems: **Windows 2000, Windows XP or higher

**• Programming languages: **MSVC++ 7.1 or higher

**• Other requirements: **None

**• License: **This is a free software. You can redistribute it and/or modify it under the terms of the GNU Lesser General Public License as published by the Free Software Foundation.

## Abbreviations

LC: Liquid Chromatography; MS: Mass Spectrometry; LTQ: Linear ion Trap Quadrupole; MALDI: Matrix Assisted Laser Desorption/Ionization; SELDI: Surface Enhanced Laser Desorption/Ionization; TOF: Time Of Flight; ICAT: Isotope Coded Affinity Tagging; ITRAQ: Isobaric Tag for Relative and Absolute Quantitation; SILAC: Stable Isotope Labeling with Amino Acids in Cell Culture; DWT: Discrete Wavelet Transform; CWT: Continuous Wavelet Transform; LSM: Local Maximum Search.

## Authors' contributions

FM, QM, YC and BL conceived the research. FM and YC implemented the software. DRG, LH, GSO and SL contributed resources and provided guidance for the research. BL, FM, QM and YC wrote and revised the manuscript. All authors read and approved the final manuscript.

## Supplementary Material

Additional file 1 **A detailed description of the wavelet transform process and the spatial Adaptive Algorithm**. A detailed description of the wavelet transform process and the spatial Adaptive Algorithm. Click here for file

Additional file 2 **Comparisons of the quantification performance of BSA in a 1:2 ratio**. Comparisons of the quantification performance of BSA mixed at 1:2 ratio between our program (Panels B) and ASAPRatio program (Panels A). Panel A and B are sepctra: OR20070625_HS_L-H-1-2_10.06981.06981.4; ions with +3 charge state. Click here for file

Additional file 3 **Comparisons of the quantification performance of BSA in a 1:4 ratio**. Comparisons of the quantification performance of BSA mixed at 1:4 ratio between our program (Panels B) and ASAPRatio program (Panels A). Panel A and B are spectra:OR20070625_HS_L-H-1-4_09.06730.06730.3; ions with +2 charge state. Click here for file

Additional file 4 **Comparisons of the quantification performance of BSA in a 4:1 ratio**. Comparisons of the quantification performance of BSA mixed at 4:1 ratio between our program (Panels B) and ASAPRatio program (Panels A). Panel A and B are spectra: OR20070625_HS_L-H-4-1.15.10440.10440.3; ions with +2 charge state. Click here for file
