# Supplementary material for: WaveletQuant, an improved quantification software based on wavelet signal threshold de-noising for labeled quantitative proteomic analysis
Source: BMC Bioinformatics. 2010 Apr 29;11:219. doi: 10.1186/1471-2105-11-219 (PMC2878310; doi:10.1186/1471-2105-11-219)
Supplement: Additional file 1 — A detailed description of the wavelet transform process and the spatial Adaptive Algorithm. A detailed description of the wavelet transform process and the spatial Adaptive Algorithm. [file 1471-2105-11-219-S1.DOC]

**Additional File 1:**

**Discrete wavelet transform（DWT）**

The wavelet transform method can be categorized as the discrete wavelet transform (DWT) or the continuous wavelet transform (CWT). Mathematically, the wavelet transform can be represented as

$WT_{f}\left(a,b\right)=\left\langle f\left(t\right),\psi_{a,b}\left(t\right)\right\rangle=\frac{1}{\sqrt{\left|a\right|}}\int_{-\infty}^{+\infty} \text{ }f\left(t\right)\text{ }\psi^{*}\left(\frac{t-b}{a}\right)\text{ }dt,$ （1）

where $f\left(t\right)$ is the signal, $a$ is the scale parameter, $b$ is the shift parameter, $\psi\left(t\right)$ is the mother wavelet.

The CWT allows wavelet transform taking the scale parameter $a$and shit parameter $b$to be any real numbers and the DWT only perform wavelet transform with integer shifts $b$and scales $a$ being the power of two. It is non-redundant, more efficient and is sufficient for exact reconstruction. As a result, the DWT is widely used in data compression and feature extraction. When we set$a_{0}=2,b_{0}=1$, then $\psi\left(t\right)$ is changed

$\psi_{j,k}\left(t\right)=2^{\frac{-j}{2}}\psi\left(2^{-j}t-k\right)$ （2）

So, the DWT coefficients can be represented as

$C_{j,k}=\int_{-\infty}^{+\infty} f\left(t\right)\psi^{*}_{j,k}\left(t\right)dt=\left\langle f,\psi_{j,k}\right\rangle$ （3）

A multi-resolution analysis consists of a sequence of successive approximation spaces$V_{j}$. More precisely, the closed subspaces$V_{j}$ satisfy

$$\cdots,\text{ }V_{0}=V_{1}\oplus W_{1},\text{ }V_{1}=V_{2}\oplus W_{2},\text{ }\cdots,\text{ }V_{j}=V_{j+1}\oplus W_{j+1},\cdots$$

The number$j$is an integer. The spaces get bigger when $j$becomes smaller. When$j=4$, we can gain the chart of spaces

Fig. S1.The decomposition of the spaces when$j=4$.

From $L^{2}\left(R\right)=\cup_{j\in Z} V_{j}$and $\text{ }V_{j-1}=V_{j}\oplus W_{j}$，we obtain

$L^{2}\left(R\right)=\oplus_{j=-\infty}^{\infty} W_{j}$ （4）

On the basis of multi-resolution analysis theory, Mallat introduces an algorithm that can quickly compute the coefficients of discrete wavelet transform. First, we put the data to high-pass filter and low-pass filter respectively to decompose the signal. Then the length of the output data is decreased to half. The output from the high-pass filter contains the detailed part of the signal, while the output from the low-pass filter contains the approximation part. We set the approximation part as the input data for the next time and then split it again and again to achieve the multi-resolution analysis of the signal. The flow chart of Mallat Algorithm is as follows:


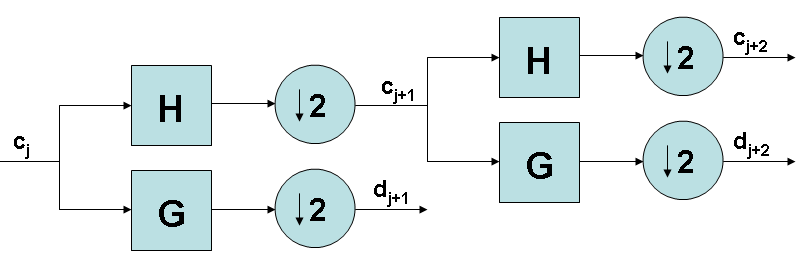


Fig. S2. The decomposition chart of Mallat Algorithm

The formula of decomposition is:

$c_{j,k}=\sum_{m} h\left(m-2k\right)c_{j-1,m}$ （5）

$d_{j,k}=\sum_{m} g\left(m-2k\right)c_{j-1,m}$ （6）

Following the same way, we can get the process of reconstruction. Its flow chart is as follows:


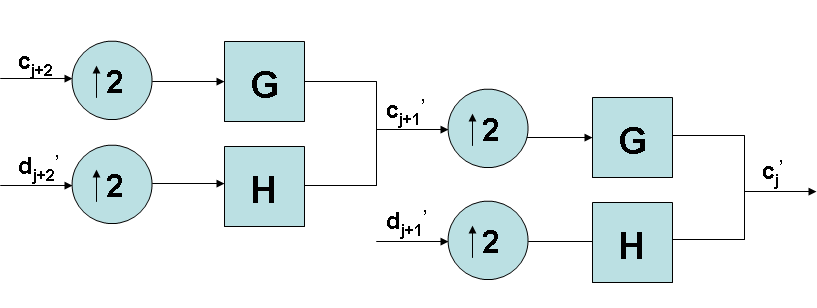


Fig. S3. The reconstruction chart of Mallat Algorithm

The formula of reconstruction is:

$c_{j-1,m}=\sum_{k} h\left(m-2k\right)c_{j,k}+\sum_{k} g\left(m-2k\right)d_{j,k}$ （7）

**Spatial Adaptive Algorithm**

Assume the largest scale of decomposition is $J$. $Wf\left(j,n\right)$ denotes DWT of signal $f$at position $n$ in scale $j$. Denote the correlation of bordered scale as follows

$Corr_{l}\left(j,n\right)=\mathop{\Pi}\limits_{i=0}^{l-1} Wf\left(j+i,n\right)$ （8）

Where $l$ represents the scale. $j<J-l+1$. As the singular of signal increases along with the increase of the scale, bordered points affect each other in the detail scale. We choose $l=2$ to compute the correlation

$Corr_{2}\left(j,n\right)=Wf\left(j,n\right)\cdot Wf\left(j+1,n\right)$ （9）

$Corr_{2}\left(j,n\right)$ is noted as correlation coefficient of the position $n$ in scale $j$.

Although wavelet coefficients are masked up by noise, their correlation coefficients strengthen magnitude along with the increasing scales, so it is easy to distinguish real signal.

To make correlation coefficient and wavelet coefficient more comparable, we define the correlation coefficient uniformly

$NewCorr_{2}\left(j,n\right)=Corr_{2}(j,n)\sqrt{P_{w}\left(j\right)/P_{Corr_{2}}\left(j\right)},\text{ }n=1,2,\cdots,N$ （10）

And

$P_{Corr_{2}}\left(j\right)=\sum_{n=1}^{N} Corr_{2}\left(j,n\right)^{2}$, $P_{w}\left(j\right)=\sum_{n=1}^{N} Wf\left(j,n\right)^{2}$ （11）

Then we compare $NewCorr_{2}\left(j,n\right)$ with $Wf\left(j,n\right)$ to obtain edges of important signals.
