# Supplementary figures and images for: WaveletQuant, an improved quantification software based on wavelet signal threshold de-noising for labeled quantitative proteomic analysis
Source: BMC Bioinformatics. 2010 Apr 29;11:219. doi: 10.1186/1471-2105-11-219 (PMC2878310; doi:10.1186/1471-2105-11-219)

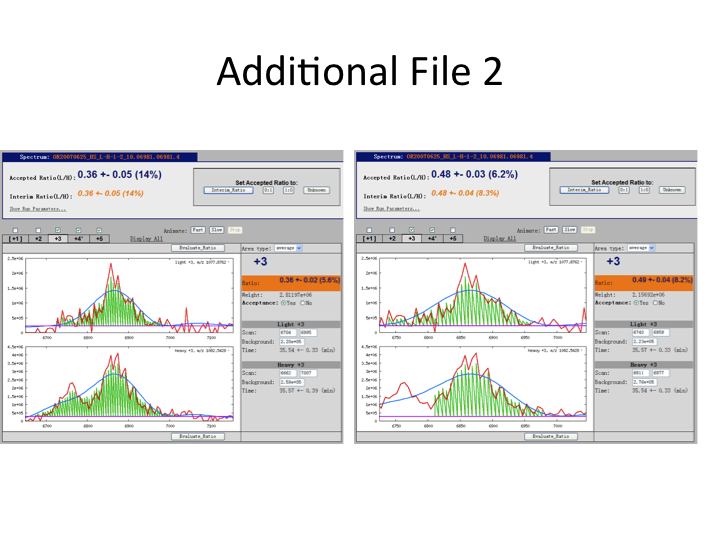

Supplement: Additional file 2 — Comparisons of the quantification performance of BSA in a 1:2 ratio. Comparisons of the quantification performance of BSA mixed at 1:2 ratio between our program (Panels B) and ASAPRatio program (Panels A). Panel A and B are sepctra: OR20070625_HS_L-H-1-2_10.06981.06981.4; ions with +3 charge state. [file 1471-2105-11-219-S2.TIFF]

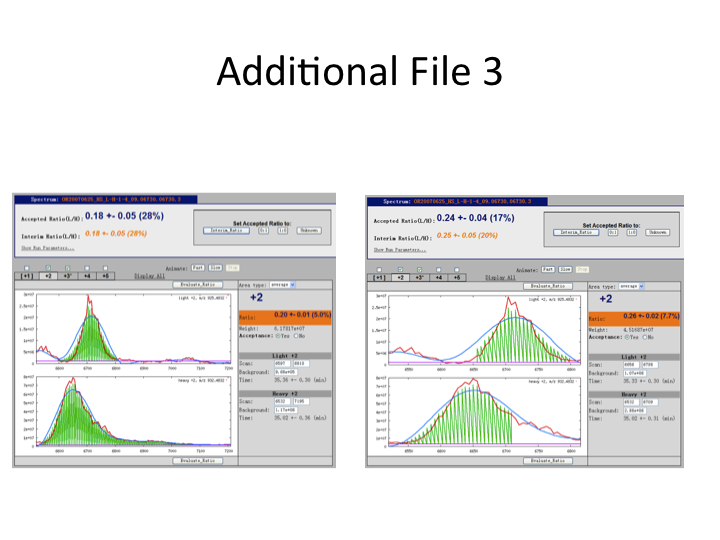

Supplement: Additional file 3 — Comparisons of the quantification performance of BSA in a 1:4 ratio. Comparisons of the quantification performance of BSA mixed at 1:4 ratio between our program (Panels B) and ASAPRatio program (Panels A). Panel A and B are spectra:OR20070625_HS_L-H-1-4_09.06730.06730.3; ions with +2 charge state. [file 1471-2105-11-219-S3.TIFF]

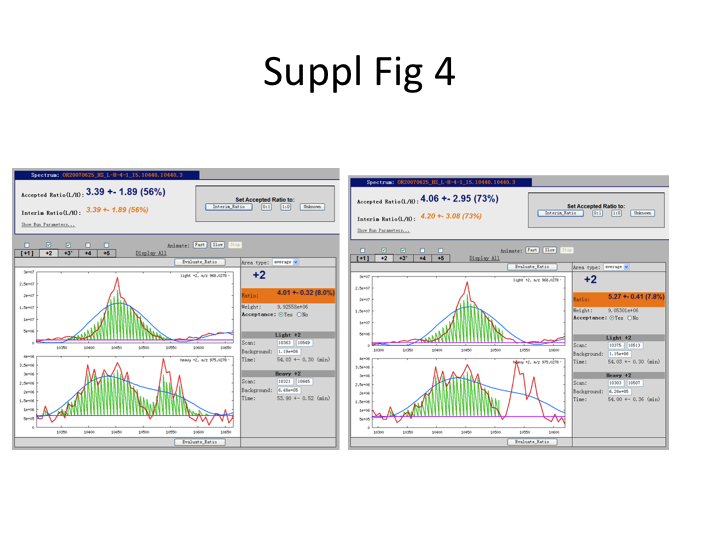

Supplement: Additional file 4 — Comparisons of the quantification performance of BSA in a 4:1 ratio. Comparisons of the quantification performance of BSA mixed at 4:1 ratio between our program (Panels B) and ASAPRatio program (Panels A). Panel A and B are spectra: OR20070625_HS_L-H-4-1.15.10440.10440.3; ions with +2 charge state. [file 1471-2105-11-219-S4.TIFF]
